# Impact of introducing procalcitonin testing on antibiotic usage in acute NHS hospitals during the first wave of COVID-19 in the UK: a controlled interrupted time series analysis of organization-level data

**DOI:** 10.1093/jac/dkac017

**Published:** 2022-02-08

**Authors:** Martin J. Llewelyn, Detelina Grozeva, Philip Howard, Joanne Euden, Sarah M. Gerver, Russell Hope, Margaret Heginbothom, Neil Powell, Colin Richman, Dominick Shaw, Emma Thomas-Jones, Robert M. West, Enitan D. Carrol, Philip Pallmann, Jonathan A. T. Sandoe

**Affiliations:** Global Health and Infectious Diseases, Brighton and Sussex Medical School, University of Sussex, Brighton, BN1 9PS, UK; Department of Microbiology and Infection, University Hospitals Sussex NHS Foundation Trust, Brighton, BN2 5BE, UK; Centre for Trials Research, Cardiff University, Neuadd Meirionnydd, Heath Park, Cardiff, CF14 4YS, UK; School of Healthcare, University of Leeds, Leeds, LS2 9JT, UK; Pharmacy Department, Leeds Teaching Hospitals, Leeds, LS1 3EX, UK; Centre for Trials Research, Cardiff University, Neuadd Meirionnydd, Heath Park, Cardiff, CF14 4YS, UK; Division of Healthcare Associated Infections and Antimicrobial Resistance, National Infection Service, Public Health England, 61 Colindale Avenue, London, NW9 5EQ, UK; Division of Healthcare Associated Infections and Antimicrobial Resistance, National Infection Service, Public Health England, 61 Colindale Avenue, London, NW9 5EQ, UK; Healthcare Associated Infection, Antimicrobial Resistance and Prescribing Programme, Public Health Wales, 2 Capital Quarter, Tyndall St, Cardiff, CF10 4BZ, UK; Pharmacy Department, Royal Cornwall Hospital Trust, Truro, TR1 3LJ, UK; Rx-Info Ltd, Exeter Science Park, 6 Babbage Way, Exeter, EX5 2FN, UK; NIHR Respiratory Biomedical Research Centre, University of Nottingham, Nottingham, NG5 1PB, UK; Centre for Trials Research, Cardiff University, Neuadd Meirionnydd, Heath Park, Cardiff, CF14 4YS, UK; University of Leeds, Worsley Building, Clarendon Way, Leeds, LS2 9LU, UK; Department of Clinical Infection, Microbiology and Immunology, University of Liverpool Institute of Infection, Veterinary and Ecological Sciences, Ronald Ross Building, 8 West Derby Street, Liverpool, L69 7BE, UK; Centre for Trials Research, Cardiff University, Neuadd Meirionnydd, Heath Park, Cardiff, CF14 4YS, UK; Department of Microbiology, The Old Medical School, The General Infirmary at Leeds, Leeds, LS1 3EX, UK

## Abstract

**Background:**

Blood biomarkers have the potential to help identify COVID-19 patients with bacterial coinfection in whom antibiotics are indicated. During the COVID-19 pandemic, procalcitonin testing was widely introduced at hospitals in the UK to guide antibiotic prescribing. We have determined the impact of this on hospital-level antibiotic consumption.

**Methods:**

We conducted a retrospective, controlled interrupted time series analysis of organization-level data describing antibiotic dispensing, hospital activity and procalcitonin testing for acute hospitals/hospital trusts in England and Wales during the first wave of COVID-19 (24 February to 5 July 2020).

**Results:**

In the main analysis of 105 hospitals in England, introduction of procalcitonin testing in emergency departments/acute medical admission units was associated with a statistically significant decrease in total antibiotic use of −1.08 (95% CI: −1.81 to −0.36) DDDs of antibiotic per admission per week per trust. This effect was then lost at a rate of 0.05 (95% CI: 0.02–0.08) DDDs per admission per week. Similar results were found specifically for first-line antibiotics for community-acquired pneumonia and for COVID-19 admissions rather than all admissions. Introduction of procalcitonin in the ICU setting was not associated with any significant change in antibiotic use.

**Conclusions:**

At hospitals where procalcitonin testing was introduced in emergency departments/acute medical units this was associated with an initial, but unsustained, reduction in antibiotic use. Further research should establish the patient-level impact of procalcitonin testing in this population and understand its potential for clinical effectiveness.

## Introduction

Identifying COVID-19 patients who have bacterial coinfection and who would benefit from antibiotic treatment is clinically challenging. Measurement of blood biomarkers of bacterial infection could help reduce unnecessary antibiotic prescribing. Biomarkers, such as C-reactive protein (CRP) and neutrophil count, are often elevated in patients with COVID-19.^1^ This, and experience of previous influenza pandemics, during which bacterial coinfection was common,^2^ has driven considerable, unnecessary antibiotic use in COVID-19 patients. In the UK, during the first wave of the pandemic, 83.1% of hospitalized patients received empiric antibiotic treatment.^3^ It is now established that bacterial coinfection is very uncommon in acute COVID-19.^4–7^ Overall volumes of antibacterial use at the beginning of the COVID-19 pandemic decreased in both primary and secondary settings, although antibacterial usage in hospital admissions increased steeply in April 2020. Use of antibacterials prescribed for respiratory infections and broad-spectrum antibacterials increased in both settings.^8^ Patients who have prolonged hospital stays, however, frequently have antibiotic-resistant nosocomial Gram-negative pathogens cultured,^9^ highlighting that early inappropriate antibiotic treatment of COVID-19 may impact on both individual patients and the wider selection of antimicrobial resistance.

Procalcitonin (PCT) is an inflammatory biomarker that rises in bacterial infection and falls in response to antibiotic treatment with greater sensitivity and specificity for bacterial infection than CRP.^10,11^ It is approved by the US FDA to support antibiotic decision-making in lower respiratory tract infection and in sepsis.^12^ Nevertheless, current US and UK national guidelines on management of community acquired pneumonia (CAP) recommend against the use of PCT to guide antibiotic prescribing.^13,14^

Early in the COVID-19 pandemic, studies reported that while substantial elevation of PCT (>0.5 ng/mL) is a feature of severe COVID-19, associated with increased mortality risk, levels in most patients are low in acute disease.^15^ During the first pandemic wave in the UK, many NHS hospitals introduced PCT testing to guide antibiotic decision-making, particularly in emergency departments (EDs) and acute medical units (AMUs).^16^ Between March and July 2020, PCT use increased from 48% to 84% of critical care units and from 11% to 51% of EDs/AMUs.^16^ This was despite COVID-19-specific guidance from NICE that PCT testing should not be used routinely in this setting.^13^

The Procalcitonin Evaluation of Antibiotic use in COVID-19 Hospitalised patients (PEACH) study^17^ is evaluating whether the use of PCT testing to guide antibiotic prescribing safely reduced antibiotic use among patients admitted to acute UK NHS hospitals with COVID-19.

Here we report the impact of PCT testing on organization-level (i.e. NHS trusts/hospitals) antibiotic use for the treatment of patients in England and Wales during the first wave of the pandemic.

## Methods

### Approvals

Research approval for the PEACH study was provided by the Health Research Authority (HRA) and Health and Care Research Wales (HCRW). Ethics approval was provided by West Midlands—Solihull Research Ethics Committee (REC Reference 21/WM/0052).

### Study design and setting

This was a retrospective controlled interrupted time series (cITS) analysis of aggregated, organization-level data. We sought to quantify the organization-level impact of introducing PCT testing on antibiotic usage during the first wave of the COVID-19 pandemic in English and Welsh hospitals, defined for the purposes of this study as ISO Weeks 9 to 27 of 2020 (24 February to 5 July).^16^ The study was designed and reported according to STROBE guidelines^18^ and additional reporting considerations specific to interrupted time series.^19^

Trusts/hospitals were categorized as follows: ‘Always Users’ if PCT testing was in use prior to the first wave of COVID-19 and continued to be used during the first wave, either in the ICU or ED/AMU or both; ‘Never Users’ if PCT testing was neither used before nor introduced during the first wave; or ‘PCT Adopters’ if PCT testing was introduced or expanded during the first wave, either in the ICU setting or among ED/AMU admissions or both.

### Variables, measures and data sources

#### Weekly antibiotic dispensing data

Weekly antibiotic dispensing data for each acute NHS trust in England and hospital in Wales were provided by Rx-Info Ltd (https://www.rx-info.co.uk/). NHS trusts were one or more hospitals under the same management. These data comprised total DDDs per week per NHS trust or hospital of all types of antibiotics dispensed to hospital locations (excluding antimycobacterial agents). DDD data were also compiled for a pre-specified subgroup of antibiotics that are used to treat CAP: amoxicillin (IV or oral), ceftriaxone (IV), cefuroxime (IV), clarithromycin (IV or oral), co-amoxiclav (IV or oral), doxycycline (oral), erythromycin (oral) and levofloxacin (IV or oral).^13,20^

#### Weekly hospital activity data

Weekly hospital activity data were provided by PHE and Public Health Wales (PHW). These included total admissions, total occupied overnight bed days, COVID-19-positive admissions and COVID-19-positive bed days per week per NHS trust (England) or hospital (Wales). COVID-19-positive admissions were defined as patients with a positive SARS-CoV-2 PCR test <14 days pre-admission or at any time during their hospital stay. Data for hospital activity in England were extracted from PHE’s source of the Secondary Use Service on 15 December 2020.^21^ Data for hospital activity recorded in patient administration systems in NHS hospitals in Wales were extracted from the Clinical Surveillance Software system ICNET on 8 January 2021.

#### PCT usage data

PCT usage data were gathered through a web-based survey as described previously.^16^ For the trusts/hospitals introducing PCT testing, their first week of PCT use was defined as the ISO week following the reported introduction date.

### Bias

We attempted to collect data from all acute NHS trusts/hospitals in England and Wales to reduce the risk of bias. Where data were excluded, we report the reason for the exclusion. Antibiotic usage data, hospital activity data and PCT usage data were collected by separate team members and data were analysed by a team who were not involved in data collection. Data collection and analysis were pre-specified in a statistical analysis plan, which can be found in the Supplementary data, available at *JAC* Online.

### Study size

The study size was determined by the number of acute NHS trusts/hospitals in England and Wales. To provide an indication of the power of this study, the following scenario was explored: there are 25 trusts who always use, 25 who never use and 50 who adopt PCT testing, 18 weeks of observations during the first wave, adopters implementing testing after 9 weeks on average, and there is an intra-class correlation coefficient (ICC) of 0.6 relating to weekly measurements within trusts. This would yield 100 × 18 observations, giving 900 with, and 900 without, PCT testing. After adjusting for clustering, this is an effective sample size of 80 + 80 and therefore a standardized effect size of 0.45, that is a medium effect size, might be estimated with 80% power. The power calculation was done in R using the function power.t.test based on a formula established by Donner *et al.*^22^

### Outcomes

The primary study outcome was total antibiotic DDDs per admission per week per NHS trust/hospital.

Secondary outcomes were: first-line CAP antibiotic DDDs (defined as above) and individual antibiotic DDDs per admission per week, and total antibiotic DDDs and CAP antibiotic DDDs per occupied overnight bed days per week per NHS trust/hospital.

### Quantitative variables and statistical analysis

The three datasets (antibiotic usage, hospital activity and PCT usage) were merged to create a single analysis dataset by matching the NHS Organisational Data Service (ODS) trust/hospital codes in the respective datasets. We anticipated that the analysis might be confounded by changes in antibiotic prescribing over time as well as changes in the number of COVID-19 admissions over time, the introduction of NICE guidance NG173^13^ and the size of trusts/hospitals, so these were all included either in the primary model or sensitivity analyses.

English and Welsh data were analysed separately because of differences in the way the NHS is organized in these countries and resulting structural differences in the data. For example, the unit of data collection in England was the NHS trust (typically comprising multiple hospitals), whereas for Wales, data were available for individual hospitals.

#### Main analysis

A cITS analysis was undertaken to estimate the organization-level effects of introducing PCT on the usage of antibiotics (normalized by trust/hospital activity), taking into account underlying trends and other covariates. To account for non-linearity of trend over time, a generalized additive mixed model (GAMM)^23^ was fitted to the data with NHS trust/hospital as a random-effect variable, allowing for variable dates of introduction of PCT testing across the trusts/hospitals.^24,25^ The GAMM included a cubic spline smoothing function as a fixed effect for time, separate fixed effects for use of PCT testing (coded 0 for no use and 1 for use in a particular week) in the ICU and ED/AMU, and their respective linear interactions with week, to assess level and/or trend changes (relative to the overall non-linear trend) in the outcome following the introduction of PCT testing. COVID-19-positive admissions as a percentage of total admissions per week per trust/hospital was included as a fixed-effect covariate. Random trust/hospital-level intercepts and slopes were included in the model to capture the variability between trusts/hospitals. The effective degrees of freedom for the smooth term were 8.8, while the number of knots used in the model was 9. Both were selected based on the default recommended by the R package ‘mgcv’. The model was checked for autocorrelation and moving averages by assessing autocorrelation and partial autocorrelation function plots by using the R package ‘forecast’.^26^

#### Additional and sensitivity analyses

Instead of modelling trend changes with interaction terms between PCT use and week, we added step effects at 4 and 8 weeks after PCT was introduced in the corresponding trusts/hospitals, to assess whether any step-change effect immediately following the introduction of PCT diminished in a non-linear way.

To assess whether the organization size had an effect on antibiotic use we included, as a measure of trust size, the publicly available 2019/20 Estates Return Information Collection (ERIC) data (data downloaded 18 May 2021; https://digital.nhs.uk/data-and-information/publications/statistical/estates-returns-information-collection).

To assess whether the introduction of NICE rapid COVID-19 guidance NG173^13^ on 1 May 2020 led to a level and/or trend change in the outcome we included a fixed effect and an interaction term with week using a binary dummy variable (0 before and 1 after the introduction date) for all trusts.

All analyses were performed in R version 4.1.0, with add-on packages ‘mgcv’ for GAMMs, ‘forecast’ for autocorrelation functions and ‘ggplot2’ for graphics. The statistical significance level was set to double-sided 5%.^27^

### Missing data

**Figure 1.**
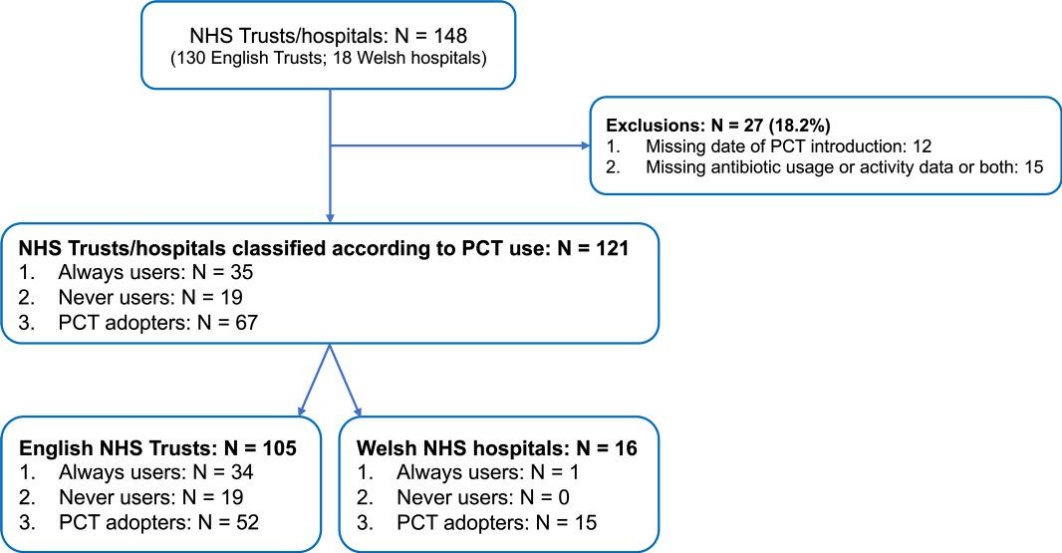
Number of NHS trusts/hospitals included in the analysis classified according to their PCT usage before and during the first wave of the COVID-19 pandemic in the UK. This figure appears in colour in the online version of *JAC* and in black and white in the print version of *JAC*.

NHS trusts/hospitals that did not provide information about their PCT usage were excluded (Figure 1). Where activity or antibiotic data were missing for a trust/hospital, these trusts/hospitals were excluded (Figure 1). The percentage of missing data is reported for all variables (DDDs and activity data) separately for the English and Welsh data (Table S1).

In addition, activity data for a total of 5 weeks for three trusts were excluded because these individual datapoints were outliers.

### Data availability statement

Data available on reasonable request from corresponding author.

## Results

Of 151 acute trusts/hospitals in England and Wales, 148 responded to the survey of PCT use.^16^ Twenty-seven were excluded from the analysis because of missing data on the date of PCT introduction, antibiotic use or hospital activity, thus leaving 121 (80%) for the final analysis (Figure 1). Based on PCT use these included: 35 ‘Always Users’ (1 in Wales); 19 ‘Never Users’ (0 in Wales); and 67 ‘PCT Adopters’ (15 in Wales). Thirty-eight NHS trusts/hospitals started using PCT in an ICU setting during the studied period, while 33 started using PCT in an ED/AMU (Figure S1). The mean and median of the elapsed weeks from the start of the studied period (24 February 2020) until the introduction of the PCT for the trusts that introduced PCT was 7.8 and 7 weeks, respectively (range: 1–18 weeks).

The variables of primary interest (DDDs per week per trust/hospital, number of admissions per week per trust/hospital, number of occupied overnight bed days per week per trust/hospital) had no missing values, while the variable for COVID-19-positive admissions had 4.4% missing data (Table S1).

**Figure 2.**
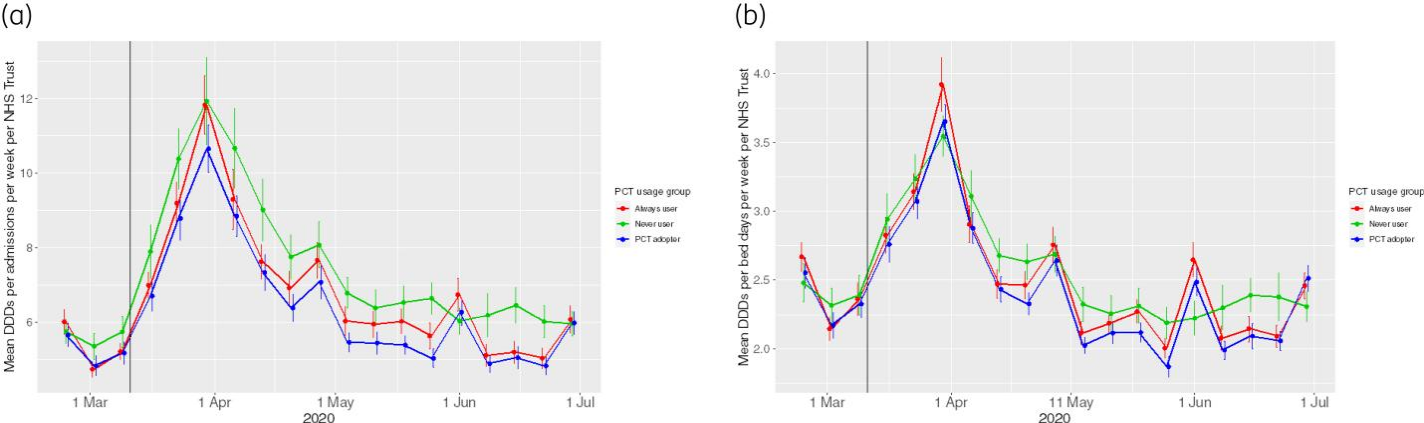
Antibiotic use at 105 NHS trusts in England during the first wave of the COVID-19 pandemic. Figures show mean antibiotic use per week per NHS trust by PCT usage. (a) Antibiotic DDDs per admission per week. (b) Mean antibiotic DDDs per occupied overnight bed days per week. The error bars in (a) and (b) show the corresponding 95% CIs. The vertical black lines represent 11 Mar 2020, when the WHO declared the novel coronavirus outbreak a global pandemic. This figure appears in colour in the online version of *JAC* and in black and white in the print version of *JAC*.

Descriptive statistics for antibiotic consumption and hospital activity data are shown in Table 1 and broken down by category of PCT usage in Table S2. Overall use of antibiotics varied 150-fold (from 188 to 28 207 DDDs per week) across different trusts and approximately 15-fold when normalized by admissions or bed days. English NHS trusts used a median of 5.9 (range: 1.7–31.3) antibiotic DDDs per admission per week, or 2.3 antibiotic DDDs per occupied overnight bed day (range: 0.5–7.3) (Table 1, Figure S2). There was also marked variation in antibiotic use over the course of the first wave of the COVID-19 pandemic, starting in late February/early March and peaking in late April (Figure 2, Figures S1–S5), reflecting the time course of hospital activity over the pandemic.

**Table 1.** Descriptive statistics for the main variables for the 121 NHS trusts/hospitals in England and Wales

Statistic	105 NHS hospital trusts in England				16 NHS hospitals in Wales			
	Mean	SD	Median	Range	Mean	SD	Median	Range
DDDs per week per trust/hospital	8427.3	4408.7	7489.7	188.1–28 207.3	4969.0	4850.6	3729.9	195.2–54 135.0
Admissions per week per trust/hospital	1445.8	869.7	1224.0	75.0–5764.0	507.5	331.1	416.0	69–1702
Occupied overnight bed days per week per trust/hospital	3490.7	1669.4	3196.0	386.0–11 027.0	2092.0	1081.7	2024.0	453–5306
COVID-19-positive admissions per week per trust/hospital	36.0	49.8	17.0	1.0–445.0	11.1	16.8	4.0	0–88.0
COVID-19-positive occupied overnight bed days per week per trust/hospital	429.9	474.7	268.0	1.0–3634.0	126.5	149.0	72.0	0–756.0
DDDs normalized by admissions per week per trust/hospital	6.6	3.1	5.9	1.7–31.3	10.7	7.0	8.9	1.9–49.9
DDDs normalized by occupied overnight bed days per week per trust/hospital	2.5	0.8	2.3	0.5–7.3	2.3	1.7	1.6	0.2–13.4

Hospitals in Wales prescribed more DDDs per admitted patient per week (median 8.9, range: 1.9–49.9) but slightly fewer per occupied overnight bed day per week per hospital (median 1.6, range: 0.2–13.4) (Table 1, Figure S5). In view of the small number of studied hospitals in Wales, and that all but one adopted PCT during the pandemic, the main analyses of the impact of PCT testing were restricted to the 105 trusts in England.

### Impact of PCT use on antibiotic consumption

**Figure 3.**
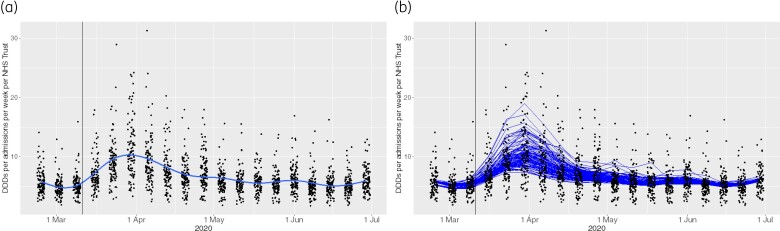
The time-course changes in antibiotic use at NHS trusts during the first wave of the COVID-19 pandemic based on the GAMM modelling. (a) Overall time trend for DDDs per admissions per week for the studied time period (24 February–5 July 2020) based on the model. (b) Fitted values for the DDDs per admission per week per NHS trust based on the model; the blue lines represent the separate 105 NHS trusts (English data). The vertical black lines represent 11 Mar 2020, when the WHO declared the novel coronavirus outbreak a global pandemic. This figure appears in colour in the online version of *JAC* and in black and white in the print version of *JAC*.

The time course of changes in antibiotic use at NHS trusts during the first wave of the pandemic modelled by the non-linear smooth term of the GAMM according to PCT is shown in Figure 3a. The results of the cITS analysis are presented in Table 2. There was no statistically significant change in the level or trend of DDDs per admission per week after PCT introduction in the ICU (*P* = 0.21). There was, however, a statistically significant decrease (*P* = 0.003) by −1.08 (95% CI: −1.81 to −0.36) DDDs per admission per week per trust immediately following the introduction of PCT in the ED/AMU, followed by a statistically significant (*P* = 0.004) increase in antibiotic prescribing of 0.05 (95% CI: 0.02–0.08) DDDs per admission per week. The variability due to trust (Figure 3b) corresponded to an ICC of 0.61.

**Table 2.** Effect sizes estimated by the cITS model of total antibiotic DDDs normalized by admissions per week per trust (English data)

	Estimate	95% CI	*P* value
Level change after PCT introduction in ICU	0.38	(−0.21 to 0.98)	0.21
Level change after PCT introduction in ED/AMU	−1.08	(−1.81 to −0.36)	0.003
Trend change after PCT introduction in ICU	−0.02	(−0.05 to 0.01)	0.21
Trend change after PCT introduction in ED/AMU	0.05	(0.02–0.08)	0.004
COVID-19-positive admissions per total admissions (%)	0.32	(0.29–0.34)	<0.001

Trend and level changes refer to deviations from the overall trend as modelled by the non-linear smooth term of the GAMM.

The effect of the percentage of COVID-19-positive admissions per total admissions each week was also highly significant (*P* < 0.001), DDDs per admission increasing by 0.32 (95% CI: 0.29–0.34) with each 1% increase in COVID-19-positive admissions, which ranged between 0% and 5% for most trusts in most weeks (Figure S4).

### Sensitivity analyses

To determine whether the introduction of NICE rapid guidance NG173 in ISO Week 19, which recommended against the use of PCT to guide antibiotic prescribing, had an effect we assessed the impact of a fixed effect and an interaction term in the model at ISO Week 19. This resulted in nearly identical estimates for both level of DDDs per admission (−1.09; 95% CI: −1.81 to −0.38; *P* = 0.003) and trend changes of DDDs per admission (0.05; 95% CI: 0.02–0.08; *P* = 0.003) after introduction of PCT testing in the ED/AMU (Table S3).

To assess the potential for non-linear increases after the initial drop in DDDs per admission following the introduction of PCT we replaced the interaction effects between PCT introduction and week with additional step-change effects at 4 and 8 weeks. This made the estimated effect sizes smaller than in the primary model and no longer statistically significant (Table S4).

When assessing the impact of trust size by adding the ERIC categories in the model, estimates of level and trend changes following PCT introduction were nearly unchanged, and there were no statistically significant differences between ERIC categories (Table S5).

As part of additional sensitivity checks, the main analysis model was run with log-transformed outcome variable and an added ARMA (2,1) term (as determined by applying the R package ‘forecast’), respectively. The two models produced similar results to the main model (Tables S6 and S7).

### Secondary outcome analyses

When repeating the main analysis but using DDDs per occupied overnight bed days per week per trust, rather than per admission, the level and trend changes following PCT introduction in the ED/AMU were smaller than those found in the model of DDDs normalized by admissions and were not statistically significant (Table S8).

Repeating the main analysis for CAP-specific antibiotics (Tables S9 and S10) again identified a statistically significant level reduction in antibiotic use followed by an upward trend when use was normalized by admissions but no statistically significant change was identified when expressed per occupied overnight bed days.

## Discussion

PCT use in 105 NHS trusts was studied for the period of the first COVID-19 wave in England (24 February to 5 July 2020). Of the 105 trusts, 34 were using PCT testing already, 19 did not introduce it in the studied period and 52 adopted PCT use in either the ICU or ED/AMU setting or in both. Using aggregated data on antibiotic use, clinical activity and PCT testing from the great majority (80%) of acute trusts in the English NHS we have demonstrated that hospitals that introduced PCT testing in the ED/AMU during the first wave of the COVID-19 pandemic experienced a drop in overall antibiotic use of approximately 1 DDD of antibiotic per admission per week. Or, expressed in another way, a trust admitting 100 patients in a week would have seen a reduction of 100 DDDs in the week after introducing PCT, compared with a trust that did not introduce PCT. This reduction was then gradually eroded over time such that, on average, it would be expected that the effect would be lost after about 20 weeks. We found a similar impact looking just at antibiotic agents that are first-line treatments for CAP, and normalizing for COVID-19 admissions rather than all admissions.

Interestingly, we found no impact of introducing PCT in the ICU. This may reflect high existing levels of antimicrobial stewardship and close working relationships between intensivists and infection specialists, which may lessen the impact of PCT testing. The analysed DDDs and hospital activity data were overall data per NHS trust/hospital and not broken down for ED/AMU or ICU. This could explain why there was no effect observed in an ICU setting.

Quantifying antibiotic use per occupied overnight bed day rather than per admission produced slightly smaller point estimates for change in level and trend than in the primary analysis but these differences were not statistically significant. This can be explained by the greater variability between trusts/hospitals in bed days than in admissions. It may also be explained by clinicians using PCT to guide decisions early in a patient’s COVID-19 admission combined with most antibiotic prescribing being initiated early in the course of the disease. When the whole of a patient’s stay is considered, as in the bed day analysis, the impact of PCT testing is diluted. Confirming this would require patient-level prescribing data linked to PCT testing. However, previous analyses of antibiotic use normalized by admissions or bed days have highlighted that the former is more accurate for acute settings with shorter length of stay, as in our analysis.^28^

We found that the initial impact of PCT testing was gradually lost over time. Of note, this is an absolute effect, not relative to other trusts/hospitals, and is likely related to sustainability, which is a challenge for any antibiotic stewardship intervention.^29^ Our finding likely reflects PCT testing being introduced without supporting aspects of a complex intervention such as a pathway for PCT use, ongoing education, audit and feedback. Again, the retrospective nature of this study means we lacked qualitative data about how PCT testing was introduced, and further research is underway within the PEACH research programme to understand this properly.

The magnitude of variation in antibiotic use we have observed appears very large, but reflects variation between trusts/hospitals, with respect to case mix (patients with COVID-19 or not) and over the time course of the pandemic. When corrected for clinical activity, the magnitude of variation falls by approximately 10-fold and is compatible with previous studies of secondary care antibiotic use across healthcare systems.^30,31^ Andrews *et al*.^8^ have recently described a marked overall reduction in secondary care antibiotic use, but a marked increase in antibiotic use per hospital admission compared with seasonal averages during the first wave of the COVID-19 pandemic. Our data for the average and range of antibiotic consumption are entirely consistent with the data they report.

In accordance with our prespecified analysis plan we have included data from NHS hospitals in Wales but excluded these from the main analysis because: (1) data were not directly comparable, being available for hospitals in Wales, but for trusts in England; (2) it became evident that there were structural differences in services in the two nations that could cause unmeasurable bias (reflected in the higher antibiotic use among Welsh hospitals and longer hospital stays for COVID-19 patients in Wales); and (3) PCT was almost universally adopted in Wales, meaning a relevant control group was not available. In addition, the natural course of the pandemic progressed differently in Wales than in England, with the number of admissions, number of occupied overnight bed days, number of COVID-19 admissions and occupied overnight bed days per week having different time courses (Figure S4).

The most important limitation of our study is its observational and opportunistic nature. PCT testing was introduced widely in the NHS in an uncoordinated and variable way and our data are subject to forms of bias that we cannot control for or fully measure. For example, regression to the mean may explain declines in antibiotic use after PCT testing was introduced. In addition, we lack any patient-level data and cannot explore aspects of implementation likely to be important in the impact of PCT (e.g. intervention fidelity). We have used data on drugs dispensed from pharmacy as a surrogate for drugs received by patients and cannot account for any drug wastage or poor compliance, thus this method may overestimate DDD usage. Data were analysed from 80% of English trusts distributed across the country and should therefore be representative, but a risk of bias caused by exclusion of some trusts is possible. Due to the nature of the available aggregated organization-level data, we were not able to test for potential confounders apart from accounting for trust size in the model. There were no statistically significant differences between the ERIC categories (Table S4). The current analysis was designed to assess the impact of PCT testing on antibiotic consumption at an organizational level. Subsequent patient-level analyses will allow for consideration of a large number of potential confounders, including many patient-level variables.

Nevertheless, these weaknesses, along with the magnitude of variation, are likely to increase the risk of our study producing a false-negative result and failing to detect an impact of PCT use on antibiotic prescribing and so it is most likely we have underestimated the true impact. At −1.08 DDDs per admission per week per trust, the impact we have detected on the level of antibiotic consumption is small but represents a reduction of approximately 18% from the national median of 5.9. For comparison, the NHS standard contract requires hospitals to achieve a 1% year-on-year reduction in total antibiotic use.^32^ Our data indicate that PCT testing has the potential to be used to reduce antibiotic overuse in COVID-19 patients. Further qualitative work and analysis of patient-level impact are needed to explore our findings further and to seek evidence for clinical effectiveness of PCT testing in this patient group.

## Supplementary Material

dkac017_Supplementary_Data

## Members of the PEACH study team

Mahableswhar ALBUR, Claire BERRY, Stuart E. BOND, Lucy BROOKES-HOWELL, Enitan D. CARROL, Paul DARK, Joanne EUDEN, Sarah M. GERVER, Thomas P. HELLYER, Josie HENLEY, Russell HOPE, Susan HOPKINS, Philip HOWARD, Martin J. LLEWELYN, Iain J. MCCULLAGH, Margaret OGDEN, Philip PALLMANN, Helena PARSONS, David G. PARTRIDGE, Neil POWELL, Colin RICHMAN, Jonathan A.T. SANDOE, Dominick SHAW, Bethany SHINKINS, Tamas SZAKMANY, Emma THOMAS-JONES, Stacy TODD and Robert M. WEST.

## Transparency declarations

J.A.T.S. has current research funding relating to diagnostic testing from NIHR, MRC, EPSRC, Wellcome Trust and Leeds Cares. Within the last 5 years, he has been involved in research funded by Pfizer, Astellas and Merck Sharp and Dohme and advised Tillots Pharma on an educational meeting. N.P. has received honoraria from Thermo Fisher. M.J.L., J.E. and P.H. are investigators on PRONTO. P.P., E.C. and E.T.J. are investigators on PRONTO, BATCH, PRECISE. C.R. is company director of Rx-info. All other authors declare no conflicts of interest.

## Supplementary data

Tables S1 to S10 and Figures S1 to S5 are available as Supplementary data at *JAC* Online.
