# Supplementary material for: Impact of introducing procalcitonin testing on antibiotic usage in acute NHS hospitals during the first wave of COVID-19 in the UK: a controlled interrupted time series analysis of organization-level data
Source: J Antimicrob Chemother. 2022 Feb 8;77(4):1189–96. doi: 10.1093/jac/dkac017 (PMC9383456; doi:10.1093/jac/dkac017)
Supplement: dkac017_Supplementary_Data [file dkac017_supplementary_data.zip › 21-1474-Supplementary data 2.pdf]

Statistical Analysis Plan for PEACH: Procalcitonin Evaluation of Antibiotic Use in COVID-19 Hospitalised Patients (Work Package 1.2 – Organisational-level Impact of PCT on Antibiotic Use)

Eudract/ISRCTN No:	n/a	Version Number:	1.0 (30/03/2021)

Final Plan

Based on protocol version: 1.1 (02/03/2021)

SAP Revision History

Protocol version	Updated SAP version no.	Section number changed	Description and reason for change	Date changed

ROLES AND RESPONSIBILITIES

**Study Statistician:** Dr Philip Pallmann, Dr Detelina Grozeva			
**Role:**			
Date:		Signature:	Via email
**Senior Statistician:** Prof Robert West			
**Role:**			
Date:		Signature:	Via email
**Chief Investigator:** Dr Jonathan Sandoe (CI)			
**Role:**			
Date:		Signature:	Via email
**Other non-signatory contributors to the SAP:** Prof Martin Llewelyn (WP Lead), Enitan Carrol (co-CI)			
**Role:**			

1. BACKGROUND

1.1 RATIONALE AND RESEARCH QUESTION

See protocol.

1.2 OBJECTIVES

To determine whether, at an NHS Trust level, having a suspected COVID-19 assessment pathway which incorporated PCT testing, modified the impact of the first COVID-19 wave on antibiotic use (both on a predefined set of antibiotics used for community-acquired pneumonia (CAP) and total antibiotics).

2. STUDY MATERIALS

2.1 STUDY DESIGN

Controlled, multi-centre, retrospective observational, aggregated (Trust level) data, interrupted time series analysis to assess the change in level and/or trend of antibiotic use, adjusted for hospital activity (e.g. admissions and bed days), following the introduction of PCT testing.

2.2 RANDOMISATION

Not applicable.

2.3 SAMPLE SIZE

Specifying a formal sample size target is not possible as this is an opportunistic retrospective analysis of antibiotic prescription data provided by our partners Rx Info Ltd, Public Health England (PHE) and Public Health Wales (PHW). Data will be available for around 100-120 English NHS Trusts and around 15-20 Welsh NHS hospitals, with up to 19 weekly data points for each.

2.4 FRAMEWORK

Retrospective analysis of routinely collected data.

2.5 INTERIM ANALYSES

2.5.1 PLANNED SAMPLE SIZE ADJUSTMENT

Not applicable.

2.5.2 STOPPING RULES

Not applicable.

2.6 TIMING OF FINAL ANALYSIS

Once all data have been made available by our partners.

2.7 TIMING OF OUTCOME ASSESSMENT

We will use aggregated data from acute admissions to Trusts between ISO weeks 9-27 (24 February 2020 to 05 July 2020 inclusive) to encompass the first peak of the COVID-19 pandemic across the UK.

3. STATISTICAL PRINCIPLES

3.1 LEVELS OF CONFIDENCE AND P-VALUES

All hypothesis tests and confidence intervals (CIs) will be two-sided. We will use a 5% significance level and present 95% CIs.

3.1.1 ADJUSTMENT FOR MULTIPLICITY

Not applicable.

3.2 ADHERENCE AND PROTOCOL DEVIATIONS

3.2.1 DEFINITION AND ASSESSMENT OF ADHERENCE

Not applicable.

3.2.2 PRESENTATION OF ADHERENCE

Not applicable.

3.2.3 DEFINITION OF PROTOCOL DEVIATION

Not applicable.

3.2.4 PRESENTATION OF PROTOCOL DEVIATIONS

Not applicable.

3.3 ANALYSIS POPULATION

To reduce the risk of bias, we will use data from all acute and teaching NHS hospital Trusts (i.e. excluding specialist hospitals) in England and Wales caring for COVID-19 inpatients over 16 years of age between 24 February 2020 and 05 July 2020. Routine hospital activity has markedly reduced during COVID-19 (e.g. cancellation of elective work) and this will have affected antibiotic prescribing and makes the pre-COVID-19 prescribing data invalid for comparison. In addition, PCT testing is expected to impact primarily on suspected COVID-19 patients who will only make up a proportion of inpatients at any one time. Hospital activity data will therefore be used to control for reduced inpatient activity.

4. STUDY POPULATION

4.1 SCREENING DATA

Not applicable.

4.2 ELIGIBILITY

Not applicable.

4.3 RECRUITMENT

Not applicable.

4.4 WITHDRAWAL/FOLLOW UP

4.4.1 LEVEL OF WITHDRAWAL

Trusts with incomplete essential information (see 4.4.3) will be fully excluded from the analysis.

4.4.2 TIMING OF WITHDRAWAL

These Trusts will be removed prior to the analysis.

4.4.3 REASONS FOR WITHDRAWAL

A Trust will be excluded if essential information about the intervention, such as the level of use of PCT testing during the first wave of COVID-19 or the date of introduction of PCT testing, cannot be obtained.

4.4.4 PRESENTATION OF WITHDRAWAL/LOSS TO FOLLOW-UP

Numbers of Trusts excluded from the analysis and reasons for exclusion will be listed in a table and/or presented in a CONSORT flow diagram.

4.5 BASELINE PARTICIPANT CHARACTERISTICS

4.5.1 LIST OF BASELINE DATA

'Baseline' data include:

- number of Trusts included in the analysis
- hospital admissions (total and COVID-positive) between ISO weeks 9 and 27 per Trust
- hospital bed days (total and COVID-positive) between ISO weeks 9 and 27 per Trust
- antibiotic use (tDDDs and CAP-DDDs, see 5.1) between ISO weeks 9 and 27 per Trust

4.5.2 DESCRIPTIVE STATISTICS

Descriptive (e.g. means or medians with standard deviations or interquartile ranges for continuous variables, frequencies and percentages for binary or categorical variables) and graphical summaries (e.g. boxplots) will be presented by country (England or Wales) and by exposure to PCT testing: 1) Trusts not using PCT (routinely) between ISO weeks 9 and 27 ('never-users'), 2) Trusts using PCT in either or both of ICU and ED/acute wards at the beginning of the first wave (ISO week 9) and continuing to do so until ISO week 27 without increasing the level of usage ('always-users'), 3) Trusts introducing PCT use for the first time or increasing PCT use (e.g. from use in ICU only to use in ICU and ED/acute wards) between ISO weeks 9 and 27 ('adopters').

5. ANALYSIS

5.1 OUTCOME DEFINITIONS

5.1.1 PRIMARY OUTCOME(S)

Weekly trend of:

- number of defined daily doses (DDDs) of all antibiotics excluding anti-TB drugs, etc. (total DDDs, 'tDDDs') per total number of admissions

5.1.2 TIMING, UNITS AND DERIVATION OF PRIMARY

Aggregated weekly antibiotic usage data (total dispensing to hospital locations) will be collected from Rx Info Ltd (and provided via PHE and PHW), and weekly patient activity data will come directly from PHE and PHW. As a consequence of the extraction and aggregation methods differing between PHE and PHW (as described in separate documents), English and Welsh data will be analysed separately.

Date of introduction of PCT testing, or not, and whether a Trust that did not introduce PCT testing between March and June 2020 was using it already or not, will be collected via direct contact with hospitals in WP 1.1. For the Trusts introducing PCT testing, their first week of PCT use will be defined as the ISO week following the reported introduction date.

The three different data elements (antibiotic usage, patient activity, PCT usage) will be merged to create a single analysis dataset; this will be done by matching their NHS Organisational Data Service (ODS) codes.

tDDDs are defined as all ATC J01, J04AB02 (rifampicin), A07AA12 (fidaxomicin) and A07AA09 (vancomycin – oral).

5.1.3 LIST OF SECONDARY OUTCOMES

Weekly trend of:

- number of prespecified antibiotics commonly used for respiratory tract infection (community-acquired pneumonia, 'CAP-DDDs') per total number of admissions
- number of tDDDs per total number of patient bed days
- number of CAP-DDDs per total number of patient bed days
- number of tDDDs per number of COVID-positive admissions
- number of CAP-DDDs per number of COVID-positive admissions
- number of tDDDs per number of COVID-positive patient bed days
- number of CAP-DDDs per number of COVID-positive patient bed days

5.1.4 ORDER OF TESTING

Not applicable.

5.1.5 TIMING, UNITS AND DERIVATION OF SECONDARIES

See 5.1.2.

CAP-DDDs are defined as amoxicillin (IV or oral), ceftriaxone (IV), cefuroxime (IV), clarithromycin (IV or oral), co-amoxiclav (IV or oral), doxycycline (oral), erythromycin (oral) and levofloxacin (IV or oral).

5.2 ANALYSIS METHODS

5.2.1 LIST OF METHODS AND PRESENTATION

A controlled interrupted time series (ITS) will be undertaken with variable dates of introduction of PCT testing across Trusts (McLintock et al. 2014; Kontopantelis et al. 2015; Bernal et al. 2017, 2018; Bottomley et al. 2019; Bell et al. 2021).

We will fit a linear mixed effects model with ISO week, use of PCT testing (yes/no) in ICU in each ISO week, use of PCT testing (yes/no) in ED/acute wards in each ISO week, and their interaction with ISO week as independent fixed-effect variables, and Trust as a random-effect variable. Based on this 'segmented' regression model, we will estimate the level and trend of the rate of antibiotic usage before and after the introduction of PCT testing and test the null hypotheses that 1) the level (i.e. model intercept) and 2) the trend (i.e. model slope) do not change following the introduction of PCT testing.

5.2.2 COVARIATE ADJUSTMENT

Not applicable.

5.2.3 ASSUMPTION CHECKING

We will check for autocorrelation by checking plots of residuals and (partial) autocorrelation functions and by performing Durbin-Watson tests.

Additionally, we will perform standard regression diagnostics (e.g. residuals vs. fitted plots) to assess basic assumptions such as linearity, normality, and homoscedasticity, as well as inspecting outliers.

5.2.4 ALTERNATIVE METHODS IF DISTRIBUTIONAL ASSUMPTIONS NOT MET

If the data show signs of autoregression, we will add a first-order autocorrelation and/or moving average covariance structure to the model.

If the trend over time in the data is distinctly non-linear, we will add additional terms (e.g. quadratic ISO week) to the regression model.

If appropriate, we will consider more complex random effects such as Trust-specific slopes in addition to Trust-specific intercepts.

5.2.5 SENSITIVITY ANALYSES

In addition to the ‘step change’ assumed for the Trusts introducing PCT testing (i.e. assuming an instant change in practice following the introduction) in the primary analysis we will also model a more gradual ‘phase-in’ e.g. over a four-week period.

We will consider adjusting for hospital occupancy (i.e. bed days divided by capacity) by including it as a covariate in the analysis model.

5.2.6 SUBGROUP ANALYSES

The outcomes defined in section 5.1 will be further subclassified, allowing us to analyse weekly trend of:

- number of DDDs of antibiotics by classification (broad spectrum, narrow spectrum)
- number of DDDs of antibiotics by route (IV, oral)
- number of DDDs of antibiotics by WHO ‘AWaRe’ category (Access, Watch, Reserve)
- number of DDDs of antibiotics by individual agent (in the list of CAPs, see 5.1.2)

per

- number of COVID-positive admissions
- total number of admissions
- number of COVID-positive patient bed days
- total number of patient bed days

Additionally, COVID-positive admissions and patient bed days will be further split into:

- admitted COVID-positive (<14 days pre-admission) or COVID-positive ≤day 7 post-admission)
- COVID-positive >day 7 post-admission

Furthermore, we will perform a separate analysis including critical care patients only.

5.3 MISSING DATA

Patterns of missingness will be explored and summarised descriptively.

5.4 ADDITIONAL ANALYSES

To assess the effects of introducing PCT in the ICU and ED/acute wards on antibiotic usage separately, we will fit an ITS model in which we replace the categorical variable describing the level of PCT testing (none, partial, full) with two separate binary variables indicating whether PCT was used or introduced in the ICU and ED/acute wards, respectively.

5.5 HARMS

Not applicable.

5.6 STATISTICAL SOFTWARE

All analyses will be performed in R version 4.0.0 or higher, with add-on packages such as 'car' for time series functions, 'nlme' for mixed effects models, and 'ggplot2' for graphics.

6. REFERENCES

6.1 NON-STANDARD STATISTICAL METHODS

Bell S, Collis R, Pallmann P, Bailey C, James K, *et al*. (2021) Reduction in massive postpartum haemorrhage and red blood cell transfusion during a national quality improvement project, Obstetric Bleeding Strategy for Wales, OBS Cymru: an observational study. Available at SSRN: https://ssrn.com/abstract=3746928

Bernal JL, Cummins S, Gasparrini A (2017) Interrupted time series regression for the evaluation of public health interventions: a tutorial. International Journal of Epidemiology, 46(1), 348-355. Corrigendum: Bernal JL, Cummins S, Gasparrini A (2020) International Journal of Epidemiology, 49(4), 1414.

Bernal JL, Cummins S, Gasparrini A (2018) The use of controls in interrupted time series studies of public health interventions. International Journal of Epidemiology, 47(6), 2082-2093.

Bottomley C, Scott JAG, Isham V (2019) Analysing interrupted time series with a control. Epidemiological Methods, 8(1), 20180010.

Kontopantelis E, Doran T, Springate DA, Buchan I, Reeves D (2015) Regression based quasi-experimental approach when randomisation is not an option: interrupted time series analysis. BMJ, 350, h2750.

McLintock K, Russell AM, Alderson SL, West R, House A, Westerman K, Foy R (2014) The effects of financial incentives for case finding for depression in patients with diabetes and coronary heart disease: interrupted time series analysis. BMJ Open, 4(8), e005178.

6.2 DATA MANAGEMENT PLAN

6.3 STUDY MASTER FILE AND STATISTICAL MASTER FILE

6.4 OTHER SOPS OR GUIDANCE DOCUMENTS

SAP DEVIATION LOG

**Document number:**		**Document version:**	
**Reason for deviation:**			
