# Supplementary material for: Impact of introducing procalcitonin testing on antibiotic usage in acute NHS hospitals during the first wave of COVID-19 in the UK: a controlled interrupted time series analysis of organization-level data
Source: J Antimicrob Chemother. 2022 Feb 8;77(4):1189–96. doi: 10.1093/jac/dkac017 (PMC9383456; doi:10.1093/jac/dkac017)
Supplement: dkac017_Supplementary_Data [file dkac017_supplementary_data.zip › 21-1474-Supplementary data.docx]

**Supplementary data**

The *Statistical analysis plan* is available as a separate Supplementary data file.

**Supplementary Tables and corresponding model equations**

Table S1: Missing data (%) for the main variables for all NHS Trusts (English data) or hospitals (Welsh data)

|  | **English data:**  **Missing data (%)** | **Welsh data:**  **Missing data (%)** |
| --- | --- | --- |
| DDDs per week per Trust/hospital | 0.0 | 0.0 |
| Admissions per week per Trust/hospital | 0.0 | 0.0 |
| Occupied overnight bed days per week per Trust/hospital | 0.0 | 0.0 |
| COVID-19 admissions per week per Trust/hospital | 4.4 | 25.9 |
| COVID-19 occupied overnight bed days per week per Trust/hospital | 1.0 | 12.0 |
| DDDs normalised by admissions per week per Trust/hospital | 0.0 | 0.0 |
| DDDs normalised by occupied overnight bed days per week per Trust/hospital | 0.0 | 0.0 |

Table S2: Descriptive statistics for the main variables for all NHS Trusts and for the NHS Trusts classified according to their PCT usage, English data

|  | **All Trusts** | | | | | **Always Users** | | | | | **Never Users** | | | | | **PCT Adopters** | | | | |
| --- | --- | --- | --- | --- | --- | --- | --- | --- | --- | --- | --- | --- | --- | --- | --- | --- | --- | --- | --- | --- |
|  | **Mean** | **SD** | **Median** | **Min** | **Max** | **Mean** | **SD** | **Median** | **Min** | **Max** | **Mean** | **SD** | **Median** | **Min** | **Max** | **Mean** | **SD** | **Median** | **Min** | **Max** |
| DDDs per week per Trust | 8427.3 | 4408.7 | 7489.7 | 188.1 | 28207.3 | 8738.5 | 4885.7 | 7250.6 | 188.1 | 28207.3 | 7722.8 | 3404.6 | 7762.9 | 1944.8 | 23146.6 | 8482.0 | 4381.8 | 7544.7 | 1280.6 | 28028.9 |
| Admissions per week per Trust | 1445.8 | 869.7 | 1224.0 | 75.0 | 5764.0 | 1486.0 | 1007.6 | 1182.5 | 75.0 | 5764.0 | 1219.4 | 715.6 | 1041.0 | 193.0 | 4047.0 | 1502.6 | 809.3 | 1298.5 | 316.0 | 4948.0 |
| Occupied overnight bed days per week per Trust | 3490.7 | 1669.4 | 3196.0 | 386.0 | 11027.0 | 3504.7 | 1768.7 | 3029.0 | 386.0 | 11027.0 | 3131.7 | 1371.0 | 3134.0 | 1008.0 | 6545.0 | 3613.2 | 1685.0 | 3309.5 | 835.0 | 10211.0 |
| COVID-19 admissions per week per Trust | 36.0 | 49.8 | 17.0 | 1.0 | 445.0 | 39.5 | 56.1 | 19.0 | 1.0 | 445.0 | 27.6 | 33.2 | 14.0 | 1.0 | 246.0 | 36.9 | 50.4 | 17.0 | 1.0 | 408.0 |
| COVID-19 occupied overnight bed days per week per Trust | 429.9 | 474.7 | 268.0 | 1.0 | 3634.0 | 455.9 | 506.0 | 278.0 | 1.0 | 3449.0 | 357.8 | 352.9 | 244.0 | 1.0 | 2208.0 | 439.4 | 490.3 | 275.5 | 1.0 | 3634.0 |
| DDDs normalised by admissions per week per Trust | 6.6 | 3.1 | 5.9 | 1.7 | 31.3 | 6.7 | 3.1 | 5.8 | 2.5 | 31.3 | 7.4 | 3.2 | 6.5 | 3.2 | 24.2 | 6.3 | 3.1 | 5.7 | 1.7 | 28.9 |
| DDDs normalised by occupied overnight bed days per week per Trust | 2.5 | 0.8 | 2.3 | 0.5 | 7.3 | 2.5 | 0.8 | 2.3 | 0.5 | 6.8 | 2.6 | 0.7 | 2.4 | 1.4 | 5.5 | 2.4 | 0.8 | 2.3 | 0.8 | 7.3 |

**Model equation for the main model. Statistical results are in Table 2 in the article.**

${(DDDs/admissions)}_{it}=\beta_{0}+f{(week)}_{t}+\beta_{1}{icu}_{it}+ \beta_{2}{edamu}_{it}+\beta_{3}{(icu:week)}_{it}+ \beta_{4}{(edamu:week)}_{it}{+ \beta}_{5}{(covid\%)}_{it}+u_{i}+\varepsilon_{it}, \varepsilon\sim N(0, \sigma^{2})$ ,

where:

- ${(DDDs/admissions)}_{it}-$ denotes total DDDs, normalised by admissions for an NHS Trust $i$ during week $t$
- $\beta_{0}-$denotes the global intercept, representing the baseline level
- $f{(week)}_{t}-$ denotes the nonlinear effect of time (cubic spline)
- $\beta_{1}-$ denotes the effect of PCT testing in ICU on total DDDs, normalised by admissions (i.e., level effect of introduction of PCT testing in ICU)
- ${icu}_{it}-$ binary covariate denoting if PCT testing was introduced in ICU by week $t$ in NHS Trust $i$
- $\beta_{2}-$denotes the effect of PCT testing in ED/AMU on total DDDs, normalised by admissions (i.e., level effect of introduction of PCT testing in ED/AMU)
- ${edamu}_{it}-$ binary covariate denoting if PCT testing was introduced in ED/AMU by week $t$ in NHS Trust $i$
- $\beta_{3}-$ indicates the slope change (i.e., trend effect) following the intervention (i.e., PCT testing) in ICU
- ${(icu:week)}_{it}-$interaction term between time and the intervention in ICU for week $t$ in NHS Trust $i$
- $\beta_{4}-$ indicates the slope change (i.e., trend effect) following the intervention (i.e., PCT testing) in ED/AMU
- ${(edamu:week)}_{it}-$interaction term between time and the intervention in ED/AMU for week $t$ in NHS Trust $i$
- $\beta_{5}-$ denotes the effect of % COVID admissions as a proportion of the total admissions in NHS Trust $i$ in week $t$
- ${(covid\%)}_{it}-$% Covid admissions as a proportion of the total admissions in NHS Trust $i$ in week $t$
- $u_{i}-$ denotes an NHS Trust random effect
- $\varepsilon_{it}-$error term

**Table S3: Effect sizes estimated by the controlled interrupted time series model of total antibiotic DDDs normalised by admissions (English data). The introduction of NICE rapid guidance NG173 in ISO week 19 (which recommended against use of PCT to guide antibiotic prescribing) is included as a covariate. Both fixed-effect and an interaction term in the model at ISO week 19 are included. Trend and level changes refer to deviations from the overall trend as modelled by the nonlinear smooth term of the generalised additive mixed model.**

|  | **Estimate** | **95% CI** | **P-value** |
| --- | --- | --- | --- |
| Level change after PCT introduction in ICU | 0.38 | (-0.20; 0.97) | 0.20 |
| Level change after PCT introduction in ED/AMU | -1.09 | (-1.81; -0.38) | 0.003 |
| Trend change after PCT introduction in ICU | -0.02 | (-0.05; 0.01) | 0.21 |
| Trend change after PCT introduction in ED/AMU | 0.05 | (-0.02; 0.08) | 0.003 |
| Level change NICE guidance | 14.55 | (-21.75; 50.86) | 0.43 |
| Trend change NICE guidance | -0.91 | (-2.86; 1.03) | 0.36 |
| % COVID-positive admissions per total admissions | 0.31 | (0.28; 0.34) | <0.001 |

CI – confidence interval; PCT – procalcitonin; ICU – Intensive Care Unit; ED/AMU – Emergency Department/Acute Medical Unit.

**Model equation for the model with NICE guidance as a covariate:**

${(DDDs/admissions)}_{it}=\beta_{0}+f{(week)}_{t}+\beta_{1}{icu}_{it}+ \beta_{2}{edamu}_{it}+\beta_{3}{(icu:week)}_{it}+ \beta_{4}\left( edamu:week \right)_{it}{+ \beta}_{5}\left( covid\% \right)_{it}{+ \beta}_{6}{nice}_{t}{+ \beta}_{7}{(nice:week)}_{t}+u_{i}+\varepsilon_{it}, \varepsilon\sim N(0, \sigma^{2})$ ,

where:

- ${(DDDs/admissions)}_{it}-$ denotes total DDDs, normalised by admissions for an NHS Trust $i$ during week $t$
- $\beta_{0}-$denotes the global intercept, representing the baseline level
- $f{(week)}_{t}-$ denotes the nonlinear effect of time (cubic spline)
- $\beta_{1}-$ denotes the effect of PCT testing in ICU on total DDDs, normalised by admissions (i.e., level effect of introduction of PCT testing in ICU)
- ${icu}_{it}-$ binary covariate denoting if PCT testing was introduced in ICU by week $t$ in NHS Trust $i$
- $\beta_{2}-$denotes the effect of PCT testing in ED/AMU on total DDDs, normalised by admissions (i.e., level effect of introduction of PCT testing in ED/AMU)
- ${edamu}_{it}-$ binary covariate denoting if PCT testing was introduced in ED/AMU by week $t$ in NHS Trust $i$
- $\beta_{3}-$ indicates the slope change (i.e., trend effect) following the intervention (i.e., PCT testing) in ICU
- ${(icu:week)}_{it}-$interaction term between time and the intervention in ICU for week $t$ in NHS Trust $i$
- $\beta_{4}-$ indicates the slope change (i.e., trend effect) following the intervention (i.e., PCT testing) in ED/AMU
- ${(edamu:week)}_{it}-$interaction term between time and the intervention in ED/AMU for week $t$ in NHS Trust $i$
- $\beta_{5}-$ denotes the effect of % COVID admissions as a proportion of the total admissions in NHS Trust $i$ in week $t$
- ${(covid\%)}_{it}-$% Covid admissions as a proportion of the total admissions in NHS Trust $i$ in week $t$
- $\beta_{6}-$denotes the effect of NICE rapid guidance NG173 (i.e., level effect of introduction of the guidance in ISO week 19)
- ${nice}_{t}-$binary covariate denoting if NICE rapid guidance NG173 was in effect in week *t*
- $\beta_{7}-$indicates the slope change (i.e., trend effect) following the introduction of NICE rapid guidance NG173
- ${(nice:week)}_{t}-$ interaction term between time and NICE rapid guidance NG173 for week $t$
- $u_{i}-$ denotes an NHS Trust random effect
- $\varepsilon_{it}-$error term

**Table S4: Effect sizes estimated by the controlled interrupted time series model of total antibiotic DDDs normalised by admissions with additional step change effects at 4 and 8 weeks (English data). Trend and level changes refer to deviations from the overall trend as modelled by the nonlinear smooth term of the generalised additive mixed model.**

|  | **Estimate** | **95% CI** | **P-value** |
| --- | --- | --- | --- |
| Level change after PCT introduction in ICU | 0.17 | (-0.18; 0.52) | 0.34 |
| Level change after PCT introduction in ED/AMU | -0.27 | (-0.64; 0.09) | 0.14 |
| Level change 4 weeks after PCT introduction in ICU | -0.31 | (-0.71; 0.09) | 0.13 |
| Level change 4 weeks after PCT introduction in ED/AMU | 0.15 | (-0.27; 0.57) | 0.49 |
| Level change 8 weeks after PCT introduction in ICU | 0.06 | (-0.36; 0.47) | 0.79 |
| Level change 8 weeks after PCT introduction in ED/AMU | 0.20 | (-0.24; 0.63) | 0.37 |
| % COVID-positive admissions per total admissions | 0.32 | (0.29; 0.34) | <0.001 |

CI – confidence interval; PCT – procalcitonin; ICU – Intensive Care Unit; ED/AMU – Emergency Department/Acute Medical Unit.

**Model equation for the model with step effects at weeks 4 and 8:**

${(DDDs/admissions)}_{it}=\beta_{0}+f{(week)}_{t}+\beta_{1}{icu}_{it}+ \beta_{2}{edamu}_{it}+{\beta_{3}(covid\%)}_{it}+{\beta_{4}{icu4w}_{it}+\beta_{5}{icu8w}_{it}+\beta_{6}{edamu4w}_{it}{+ \beta}_{7}{edamu8w}_{it}+u}_{i}+\varepsilon_{it}, \varepsilon\sim N(0, \sigma^{2})$ ,

where:

- ${(DDDs/admissions)}_{it}-$ denotes total DDDs, normalised by admissions for an NHS Trust $i$ during week $t$
- $\beta_{0}-$denotes the global intercept, representing the baseline level
- $f{(week)}_{t}-$ denotes the nonlinear effect of time (cubic spline)
- $\beta_{1}-$ denotes the effect of PCT testing in ICU on total DDDs, normalised by admissions (i.e., level effect of introduction of PCT testing in ICU)
- ${icu}_{it}-$ binary covariate denoting if PCT testing was introduced in ICU by week $t$ in NHS Trust $i$
- $\beta_{2}-$denotes the effect of PCT testing in ED/AMU on total DDDs, normalised by admissions (i.e., level effect of introduction of PCT testing in ED/AMU)
- ${edamu}_{it}-$ binary covariate denoting if PCT testing was introduced in ED/AMU by week $t$ in NHS Trust $i$
- $\beta_{3}-$ denotes the effect of % COVID admissions as a proportion of the total admissions in NHS Trust $i$ in week $t$
- $\beta_{4}, \beta_{5}, \beta_{6}, \beta_{7}-$denotes the delayed effect in ICU and ED/AMU 4 weeks or 8 weeks after the introduction of PCT testing
- ${icu4w}_{it}, {icu8w}_{it}, {edamu4w}_{it}, {edamu4w}_{it}-$binary covariate denoting 4 weeks or 8 weeks after the PCT testing was introduced in ICU or ED/AMU by week $t$ in NHS Trust $i$
- ${(covid\%)}_{it}-$% Covid admissions as a proportion of the total admissions in NHS Trust $i$ in week $t$
- $u_{i}-$ denotes an NHS Trust random effect
- $\varepsilon_{it}-$error term

**Table S5: Effect sizes estimated by the controlled interrupted time series model of total antibiotic DDDs normalised by admissions with Trust size as an additional covariate (English data). The reference category is “Trust type: acute – large”. Trend and level changes refer to deviations from the overall trend as modelled by the nonlinear smooth term of the generalised additive mixed model.**

|  | **Estimate** | **95% CI** | **P-value** |
| --- | --- | --- | --- |
| Level change after PCT introduction in ICU | 0.37 | (-0.22; 0.98) | 0.22 |
| Level change after PCT introduction in ED/AMU | -1.07 | (-1.79; -0.35) | 0.004 |
| Trend change after PCT introduction in ICU | -0.02 | (-0.05; 0.01) | 0.21 |
| Trend change after PCT introduction in ED/AMU | 0.05 | (0.02; 0.08) | 0.004 |
| % COVID-positive admissions per total admissions | 0.31 | (0.29; 0.34) | <0.001 |
| Trust type: acute – medium | 0.27 | (-0.78; 1.33) | 0.61 |
| Trust type: acute – multiservice | 1.03 | (-1.22; 3.28) | 0.37 |
| Trust type: acute – small | 0.04 | (-1.00; 3.28) | 0.93 |
| Trust type: acute – teaching | -0.47 | (-1.47; 0.53) | 0.36 |

CI – confidence interval; PCT – procalcitonin; ICU – Intensive Care Unit; ED/AMU – Emergency Department/Acute Medical Unit.

**Model equation for the model with NHS Trust size based on ERIC data from NHS Digital:**

${(DDDs/admissions)}_{it}=\beta_{0}+f{(week)}_{t}+\beta_{1}{icu}_{it}+ \beta_{2}{edamu}_{it}+\beta_{3}{(icu:week)}_{it}+ \beta_{4}{(edamu:week)}_{it}{+ \beta}_{5}{(covid\%)}_{it}{+ \beta}_{6}{eric}_{i}+u_{i}+\varepsilon_{it}, \varepsilon\sim N(0, \sigma^{2})$ ,

where:

- ${(DDDs/admissions)}_{it}-$ denotes total DDDs, normalised by admissions for an NHS Trust $i$ during week $t$
- $\beta_{0}-$denotes the global intercept, representing the baseline level
- $f{(week)}_{t}-$ denotes the nonlinear effect of time (cubic spline)
- $\beta_{1}-$ denotes the effect of PCT testing in ICU on total DDDs, normalised by admissions (i.e., level effect of introduction of PCT testing in ICU)
- ${icu}_{it}-$ binary covariate denoting if PCT testing was introduced in ICU by week $t$ in NHS Trust $i$
- $\beta_{2}-$denotes the effect of PCT testing in ED/AMU on total DDDs, normalised by admissions (i.e., level effect of introduction of PCT testing in ED/AMU)
- ${edamu}_{it}-$ binary covariate denoting if PCT testing was introduced in ED/AMU by week $t$ in NHS Trust $i$
- $\beta_{3}-$ indicates the slope change (i.e., trend effect) following the intervention (i.e., PCT testing) in ICU
- ${(icu:week)}_{it}-$interaction term between time and the intervention in ICU for week $t$ in NHS Trust $i$
- $\beta_{4}-$ indicates the slope change (i.e., trend effect) following the intervention (i.e., PCT testing) in ED/AMU
- ${(edamu:week)}_{it}-$interaction term between time and the intervention in ED/AMU for week $t$ in NHS Trust $i$
- $\beta_{5}-$ denotes the effect of % COVID admissions as a proportion of the total admissions in NHS Trust $i$ in week $t$
- ${(covid\%)}_{it}-$% Covid admissions as a proportion of the total admissions in NHS Trust $i$ in week $t$
- $\beta_{6}-$ denotes the effect of NHS Trust size (based on ERIC categories)
- ${eric}_{i}-$ denotes the Trust size (based on ERIC categories) for NHS Trust $i$
- $u_{i}-$ denotes an NHS Trust random effect
- $\varepsilon_{it}-$error term

**Table S6: Effect sizes estimated by the controlled interrupted time series model of DDDs normalised by admissions (English data), log-transformed. Trend and level changes refer to deviations from the overall trend as modelled by the nonlinear smooth term of the generalised additive mixed model.**

|  | **Estimate** | **95% CI** | **P-value** |
| --- | --- | --- | --- |
| Level change after PCT introduction in ICU | 0.06 | (-0.02; 0.13) | 0.16 |
| Level change after PCT introduction in ED/AMU | -0.15 | (-0.24; -0.05) | 0.002 |
| Trend change after PCT introduction in ICU | -0.004 | (-0.01; 0.00) | 0.13 |
| Trend change after PCT introduction in ED/AMU | 0.01 | (0.00; 0.01) | 0.004 |
| % COVID-positive admissions per total admissions | 0.02 | (0.02; 0.02) | <0.001 |

CI – confidence interval; PCT – procalcitonin; ICU – Intensive Care Unit; ED/AMU – Emergency Department/Acute Medical Unit.

**Model equation for the model with a log-transformed dependent variable (DDDs normalised by admissions):**

${log(DDDs/admissions)}_{it}=\beta_{0}+f{(week)}_{t}+\beta_{1}{icu}_{it}+ \beta_{2}{edamu}_{it}+\beta_{3}{(icu:week)}_{it}+ \beta_{4}{(edamu:week)}_{it}{+ \beta}_{5}{(covid\%)}_{it}+u_{it}+\varepsilon_{it}, \varepsilon\sim N(0, \sigma^{2})$ ,

where:

- ${log(DDDs/admissions)}_{it}-$ denotes total DDDs, normalised by admissions for an NHS Trust $i$ during week $t$, log-transformed
- $\beta_{0}-$denotes the global intercept, representing the baseline level
- $f{(week)}_{t}-$ denotes the nonlinear effect of time (cubic spline)
- $\beta_{1}-$ denotes the effect of PCT testing in ICU on total DDDs, normalised by admissions (i.e., level effect of introduction of PCT testing in ICU)
- ${icu}_{it}-$ binary covariate denoting if PCT testing was introduced in ICU by week $t$ in NHS Trust $i$
- $\beta_{2}-$denotes the effect of PCT testing in ED/AMU on total DDDs, normalised by admissions (i.e., level effect of introduction of PCT testing in ED/AMU)
- ${edamu}_{it}-$ binary covariate denoting if PCT testing was introduced in ED/AMU by week $t$ in NHS Trust $i$
- $\beta_{3}-$ indicates the slope change (i.e., trend effect) following the intervention (i.e., PCT testing) in ICU
- ${(icu:week)}_{it}+ -$interaction term between time and the intervention in ICU for week $t$ in NHS Trust $i$
- $\beta_{4}-$ indicates the slope change (i.e., trend effect) following the intervention (i.e., PCT testing) in ED/AMU
- ${(edamu:week)}_{it}+ -$interaction term between time and the intervention in ED/AMU for week $t$ in NHS Trust $i$
- $\beta_{5}-$ denotes the effect of % COVID admissions as a proportion of the total admissions in NHS Trust $i$ in week $t$
- ${(covid\%)}_{it}-$% Covid admissions as a proportion of the total admissions in NHS Trust $i$ in week $t$
- $u_{i}-$ denotes an NHS Trust random effect
- $\varepsilon_{it}-$error term

**Table S7: Effect sizes estimated by the controlled interrupted time series model of DDDs normalised by admissions (English data). Autoregression moving average ARMA(2,1) is included in the model. Trend and level changes refer to deviations from the overall trend as modelled by the nonlinear smooth term of the generalised additive mixed model.**

|  | **Estimate** | **95% CI** | **P-value** |
| --- | --- | --- | --- |
| Level change after PCT introduction in ICU | 0.35 | (-0.32; 1.02) | 0.31 |
| Level change after PCT introduction in ED/AMU | -1.14 | (-1.95; -0.33) | 0.01 |
| Trend change after PCT introduction in ICU | -0.02 | (-0.06; 0.02) | 0.26 |
| Trend change after PCT introduction in ED/AMU | 0.05 | (0.01; 0.09) | 0.01 |
| % COVID-positive admissions per total admissions | 0.31 | (0.28; 0.34) | <0.001 |

CI – confidence interval; PCT – procalcitonin; ICU – Intensive Care Unit; ED/AMU – Emergency Department/Acute Medical Unit.

**Model equation with included autoregressive moving average:** The model is the same as the main model with included correlation structure for the error term (ARMA(2,1)).

**Table S8: Effect sizes estimated by the controlled interrupted time series model of total antibiotic DDDs normalised by occupied overnight bed days (English data). Trend and level changes refer to deviations from the overall trend as modelled by the nonlinear smooth term of the generalised additive mixed model.**

|  | **Estimate** | **95% CI** | **P-value** |
| --- | --- | --- | --- |
| Level change after PCT introduction in ICU | 0.04 | (-0.15; 0.23) | 0.681 |
| Level change after PCT introduction in ED/AMU | -0.14 | (-0.37; 0.09) | 0.234 |
| Trend change after PCT introduction in ICU | -0.0004 | (-0.01; 0.01) | 0.946 |
| Trend change after PCT introduction in ED/AMU | 0.005 | (-0.01; 0.02) | 0.414 |
| % COVID-positive admissions per total admissions | 0.02 | (0.02; 0.03) | <0.001 |

CI – confidence interval; PCT – procalcitonin; ICU – Intensive Care Unit; ED/AMU – Emergency Department/Acute Medical Unit.

**Model equation for the model with dependent variable: DDDs normalised by occupied overnight bed days:**

${(DDDs/beddays)}_{it}=\beta_{0}+f{(week)}_{t}+\beta_{1}{icu}_{it}+ \beta_{2}{edamu}_{it}+\beta_{3}{(icu:week)}_{it}+ \beta_{4}{(edamu:week)}_{it}{+ \beta}_{5}{(covid\%)}_{it}+u_{i}+\varepsilon_{it}, \varepsilon\sim N(0, \sigma^{2})$ ,

where:

- ${(DDDs/beddays)}_{it}-$ denotes total DDDs, normalised by bed days for an NHS Trust $i$ during week $t$
- $\beta_{0}-$denotes the global intercept, representing the baseline level
- $f{(week)}_{t}-$ denotes the nonlinear effect of time (cubic spline)
- $\beta_{1}-$ denotes the effect of PCT testing in ICU on total DDDs, normalised by admissions (i.e., level effect of introduction of PCT testing in ICU)
- ${icu}_{it}-$ binary covariate denoting if PCT testing was introduced in ICU by week $t$ in NHS Trust $i$
- $\beta_{2}-$denotes the effect of PCT testing in ED/AMU on total DDDs, normalised by admissions (i.e., level effect of introduction of PCT testing in ED/AMU)
- ${edamu}_{it}-$ binary covariate denoting if PCT testing was introduced in ED/AMU by week $t$ in NHS Trust $i$
- $\beta_{3}-$ indicates the slope change (i.e., trend effect) following the intervention (i.e., PCT testing) in ICU
- ${(icu:week)}_{it}-$interaction term between time and the intervention in ICU for week $t$ in NHS Trust $i$
- $\beta_{4}-$ indicates the slope change (i.e., trend effect) following the intervention (i.e., PCT testing) in ED/AMU
- ${(edamu:week)}_{it}-$interaction term between time and the intervention in ED/AMU for week $t$ in NHS Trust $i$
- $\beta_{5}-$ denotes the effect of % COVID admissions as a proportion of the total admissions in NHS Trust $i$ in week $t$
- ${(covid\%)}_{it}-$% Covid admissions as a proportion of the total admissions in NHS Trust $i$ in week $t$
- $u_{i}-$ denotes an NHS Trust random effect
- $\varepsilon_{it}-$error term

**Table S9: Effect sizes estimated by the controlled interrupted time series model of CAP DDDs normalised by admissions (English data). Trend and level changes refer to deviations from the overall trend as modelled by the nonlinear smooth term of the generalised additive mixed model.**

|  | **Estimate** | **95% CI** | **P-value** |
| --- | --- | --- | --- |
| Level change after PCT introduction in ICU | 0.17 | (-0.23; 0.56) | 0.42 |
| Level change after PCT introduction in ED/AMU | -0.67 | (-1.16; -0.19) | 0.01 |
| Trend change after PCT introduction in ICU | -0.01 | (-0.03; 0.01) | 0.34 |
| Trend change after PCT introduction in ED/AMU | 0.03 | (0.01; 0.05) | 0.01 |
| % COVID-positive admissions per total admissions | 0.25 | (0.24; 0.27) | <0.001 |

CI – confidence interval; PCT – procalcitonin; ICU – Intensive Care Unit; ED/AMU – Emergency Department/Acute Medical Unit.

**Model equation for the model with dependent variable: CAP DDDs normalised by admissions:**

${(CAP DDDs/admissions)}_{it}=\beta_{0}+f{(week)}_{t}+\beta_{1}{icu}_{it}+ \beta_{2}{edamu}_{it}+\beta_{3}{(icu:week)}_{it}+ \beta_{4}{(edamu:week)}_{it}{+ \beta}_{5}{(covid\%)}_{it}+u_{i}+\varepsilon_{it}, \varepsilon\sim N(0, \sigma^{2})$ ,

where:

- ${(CAP DDDs/admissions)}_{it}-$ denotes CAP DDDs, normalised by admissions for an NHS Trust $i$ during week $t$
- $\beta_{0}-$denotes the global intercept, representing the baseline level
- $f{(week)}_{t}-$ denotes the nonlinear effect of time (cubic spline)
- $\beta_{1}-$ denotes the effect of PCT testing in ICU on total DDDs, normalised by admissions (i.e., level effect of introduction of PCT testing in ICU)
- ${icu}_{it}-$ binary covariate denoting if PCT testing was introduced in ICU by week $t$ in NHS Trust $i$
- $\beta_{2}-$denotes the effect of PCT testing in ED/AMU on total DDDs, normalised by admissions (i.e., level effect of introduction of PCT testing in ED/AMU)
- ${edamu}_{it}-$ binary covariate denoting if PCT testing was introduced in ED/AMU by week $t$ in NHS Trust $i$
- $\beta_{3}-$ indicates the slope change (i.e., trend effect) following the intervention (i.e., PCT testing) in ICU
- ${(icu:week)}_{it}+ -$interaction term between time and the intervention in ICU for week $t$ in NHS Trust $i$
- $\beta_{4}-$ indicates the slope change (i.e., trend effect) following the intervention (i.e., PCT testing) in ED/AMU
- ${(edamu:week)}_{it}+ -$interaction term between time and the intervention in ED/AMU for week $t$ in NHS Trust $i$
- $\beta_{5}-$ denotes the effect of % COVID admissions as a proportion of the total admissions in NHS Trust $i$ in week $t$
- ${(covid\%)}_{it}-$% Covid admissions as a proportion of the total admissions in NHS Trust $i$ in week $t$
- $u_{i}-$ denotes an NHS Trust random effect
- $\varepsilon_{it}-$error term

**Table S10: Effect sizes estimated by the controlled interrupted time series model of CAP DDDs normalised by occupied overnight bed days (English data). Trend and level changes refer to deviations from the overall trend as modelled by the nonlinear smooth term of the generalised additive mixed model.**

|  | **Estimate** | **95% CI** | **P-value** |
| --- | --- | --- | --- |
| Level change after PCT introduction in ICU | 0.01 | (-0.11; 0.13) | 0.87 |
| Level change after PCT introduction in ED/AMU | -0.09 | (-0.25; 0.06) | 0.22 |
| Trend change after PCT introduction in ICU | -0.0003 | (-0.01; 0.01) | 0.93 |
| Trend change after PCT introduction in ED/AMU | 0.003 | (-0.004; 0.01) | 0.46 |
| % COVID-positive admissions per total admissions | 0.03 | (0.03; 0.04) | <0.001 |

CI – confidence interval; PCT – procalcitonin; ICU – Intensive Care Unit; ED/AMU – Emergency Department/Acute Medical Unit.

**Model equation for the model with dependent variable: CAP DDDs normalised by occupied overnight bed days:**

${(CAP DDDs/beddays)}_{it}=\beta_{0}+f{(week)}_{t}+\beta_{1}{icu}_{it}+ \beta_{2}{edamu}_{it}+\beta_{3}{(icu:week)}_{it}+ \beta_{4}{(edamu:week)}_{it}{+ \beta}_{5}{(covid\%)}_{it}+u_{i}+\varepsilon_{it}, \varepsilon\sim N(0, \sigma^{2})$ ,

where:

- ${(CAP DDDs/beddays)}_{it}-$ denotes CAP DDDs, normalised by occupied overnight bed days for an NHS Trust $i$ during week $t$
- $\beta_{0}-$denotes the global intercept, representing the baseline level
- $f{(week)}_{t}-$ denotes the nonlinear effect of time (cubic spline)
- $\beta_{1}-$ denotes the effect of PCT testing in ICU on total DDDs, normalised by admissions (i.e., level effect of introduction of PCT testing in ICU)
- ${icu}_{it}-$ binary covariate denoting if PCT testing was introduced in ICU by week $t$ in NHS Trust $i$
- $\beta_{2}-$denotes the effect of PCT testing in ED/AMU on total DDDs, normalised by admissions (i.e., level effect of introduction of PCT testing in ED/AMU)
- ${edamu}_{it}-$ binary covariate denoting if PCT testing was introduced in ED/AMU by week $t$ in NHS Trust $i$
- $\beta_{3}-$ indicates the slope change (i.e., trend effect) following the intervention (i.e. PCT testing) in ICU
- ${(icu:week)}_{it}-$interaction term between time and the intervention in ICU for week $t$ in NHS Trust $i$
- $\beta_{4}-$ indicates the slope change (i.e., trend effect) following the intervention (i.e. PCT testing) in ED/AMU
- ${(edamu:week)}_{it}-$interaction term between time and the intervention in ED/AMU for week $t$ in NHS Trust $i$
- $\beta_{5}-$ denotes the effect of % COVID admissions as a proportion of the total admissions in NHS Trust $i$ in week $t$
- ${(covid\%)}_{it}-$% Covid admissions as a proportion of the total admissions in NHS Trust $i$ in week $t$
- $u_{i}-$ denotes an NHS Trust random effect
- $\varepsilon_{it}-$error term

**Figure S1:** DDDs per admissions per week per NHS Trust for all NHS Trusts separately. The introduction of PCT testing in the relevant NHS Trusts, which started to use the test during the 1^st^ COVID-19 wave is depicted with change of colour

**Figure S2:** Mean of the main variables during the first wave of the COVID-19 pandemic for the studied NHS Trusts (English data)

(a) DDDs per week per NHS Trust (b) Number of admissions per week per NHS Trust (c) Number of occupied overnight bed days per week per NHS Trust (d) Number of COVID-19 admissions per week per NHS Trust

| (a) | (b) |
| --- | --- |
|  |  |
| (c) | (d) |
|  |  |

11 Mar 2020: The WHO declares the novel coronavirus outbreak a global pandemic

**Figure S3**: Violin plots for the overall antibiotic use (DDDs) and DDDs normalised by admission for the studied NHS Trusts (English data)

(a) Overall antibiotic use (DDDs) per week by PCT user status (b) Overall DDDs normalised by admissions per week per NHS Trust by PCT user status

| (a) | (b) |
| --- | --- |
|  |  |

**Figure S4:** Main variables during the first wave of the COVID-19 pandemic for the studied NHS Trusts (English data) presented as one line per NHS Trust

(a) DDDs per week per NHS Trust (b) Number of admissions per week per NHS Trust (c) Number of occupied overnight bed days per week per NHS Trust (d) Number of COVID-19 admissions per week per NHS Trust

| (a) | (b) |
| --- | --- |
|  |  |
| (c) | (d) |
|  |  |

11 Mar 2020: The WHO declares the novel coronavirus outbreak a global pandemic

**Figure S5:** Main variables during the first wave of the COVID-19 pandemic for the studied NHS Trusts (English data) and the Welsh hospitals

(a) Mean DDDs normalised by admissions per week per NHS Trust/hospital (b) Mean DDDs normalised by occupied overnight bed days per week per NHS Trust/hospital (c) Mean DDDs normalised by COVID-19 admissions per week per NHS Trust/hospital (d) Mean DDDs normalised by occupied overnight COVID-19 bed days per week per NHS Trust/hospital

| (a) | (b) |
| --- | --- |
|  |  |
| (c) | (d) |
|  |  |

11 Mar 2020: The WHO declares the novel coronavirus outbreak a global pandemic
